# Quantifying the effect of glucose 6-phosphate dehydrogenase deficiency on glycated hemoglobin values in children and adolescents with type 1 diabetes

**DOI:** 10.1038/s41598-024-57958-3

**Published:** 2024-03-27

**Authors:** Carlo Ripoli, Maria Rossella Ricciardi, Maria Rosaria Angelo, Daniela Ripoli

**Affiliations:** 1 Pediatric Diabetology Unit, ASL 8 Cagliari, Cagliari, Sardinia Italy; 2 Department of Life and Environmental Sciences, School of Specialization in Hospital Pharmacy, University of Cagliari, Cagliari, Italy

**Keywords:** Type 1 diabetes, G6PD deficiency, Glycated hemoglobin, Children, Adolescents, Endocrinology, Endocrine system and metabolic diseases

## Abstract

The primary objectives of the study were (a) to confirm that glucose 6-phosphate dehydrogenase (G6PD) deficiency affects HbA1c values in a sample of children and adolescents with type 1 diabetes (T1D) and (b) to quantify this effect so that a correction can be applied to the HbA1c values found in current clinical practice. The following data were collected: age, sex, G6PD, number of daily capillary blood glucose measurements, 90-day average blood glucose levels prior to the study, HbA1c, and glycated hemoglobin estimated (eA1c) obtained from blood glucose levels. Patients were divided into three groups based on G6PD values: deficient, intermediate, and nondeficient. In each group, a comparison between the average eA1C and HbA1c values was performed. Then, the difference between the eA1c and HbA1c values of each patient and the mean of the differences (MD) of all patients was calculated within the three groups. Finally, a comparison of the MD values between groups was performed. Seventy-four subjects with T1D were studied. Based on the G6PD value, 33 subjects were deficient, 8 were intermediate, and 33 subjects were nondeficient. In deficient patients, the eA1c values were significantly higher than the HbA1c values. In the other two groups, however, there were no differences. The MD values between the three groups were significantly different. In deficient patients, MD values were higher than those in intermediate and in nondeficient patients. No difference was found between intermediate and nondeficient subjects. Our study confirms that G6PD deficiency affects HbA1c values in children and adolescents with T1D, both in deficient subjects and, to a much lesser extent, in intermediate subjects. In deficient subjects, there is an average reduction in HbA1c attributable to enzyme deficiency of 1.3% (14 mmol/mol) and in intermediate subjects of 0.3% (3 mmol/mol).

## Introduction

Type 1 diabetes (T1D) is a chronic condition characterized by a deficit in insulin secretion secondary to immune-mediated damage to pancreatic beta cells. It occurs in genetically predisposed individuals and requires lifelong insulin replacement therapy^1^. Optimal metabolic control is necessary from the onset of the disease to prevent chronic microvascular complications (retinopathy, nephropathy, neuropathy)^2,3^.

Despite the widespread use of sensors for monitoring subcutaneous glucose and the standardization of the metrics collected in the ambulatory glucose profile (AGP), glycated hemoglobin (HbA1c) remains an important parameter for monitoring metabolic control in patients with T1D^4^. Several factors can cause falsely elevated or reduced HbA1c values and one of these is the deficiency of the enzyme glucose 6-phosphate dehydrogenase (G6PD)^5^. The G6PD gene is located on the long arm of the X chromosome (Xq28 band) and is transmitted according to the mechanisms of X-linked inheritance. There are more than 200 G6PD variants, distinguished by biochemical and functional characteristics that are associated with a different susceptibility to oxidative/hemolytic stimuli. The most frequent variants are the African or GdA- and the Mediterranean GdB-. In the latter, erythrocyte enzyme activity determined in the laboratory is practically absent (0–5%)^6^. In males there are only 2 genotypes: hemizygous nondeficient (normal) and hemizygous deficient. In females there are 3 genotypes: homozygous nondeficient (normal), homozygous deficient, and heterozygous with intermediate values of enzyme^7^. G6PD deficiency can cause several clinical manifestations including hemolytic anemia following fava bean ingestion (favism), drugs, and infections. It may also be responsible for neonatal jaundice and chronic nonspherocytic hemolytic anemia^8,9^. In children and adolescents at the onset of type 1 diabetes, G6PD deficiency can lead to acute hemolytic anemia. This usually occurs a few days after the start of insulin therapy when normalizing blood sugar levels and sometimes requires transfusion therapy^10,11^.

Several studies have shown that subjects with T1D and G6PD deficiency have lower HbA1c values than those who are not deficient^12–14^. In particular, Meloni et al.^15^ found significantly lower Hba1c values in both healthy subjects and T1D patients in the deficient group than in the nondeficient group. However, there are no studies that have quantified the relationship between G6PD deficiency and HbA1c values in clinical practice, either in the follow-up of T1D patients or in patients with occasional hyperglycemia (prediabetes).

The primary objectives of the study were: (a) to confirm that G6PD deficiency affects HbA1c values in a sample of children and adolescents with T1D, and (b) to quantify this effect so that a correction can be applied to the HbA1c values found in current clinical practice.

The secondary objective was to evaluate whether the difference between the estimated glycated hemoglobin (eA1c) and the laboratory glycated hemoglobin (HbA1c) was influenced by the value of the latter and by the age of patients.

## Methods

This is a monocentric observational study with a retrospective data collection regarding patients followed in the period from January 2019 until December 2020, preceding the widespread use of sensors for subcutaneous glucose monitoring in subjects with T1D of pediatric age in Sardinia. We included patients with T1D who were followed in the Pediatric Diabetology Unit of the Microcythemic Hospital of Cagliari (Sardinia) and met the following criteria:

- Sardinian origin
- MCV values on blood count > 80 fL
- At least 4 capillary blood glucose measurements per day in the 90 days preceding the investigation
- Use of a glucometer for measuring capillary blood glucose levels with high accuracy (mean absolute relative difference, MARD < 6%)

Subjects with other chronic diseases (genetic, haematological, etc.) that can interfere with blood count values were excluded. In all participants, the following data were collected: age, sex, G6PD, number of daily capillary blood glucose measurements, 90-day average blood glucose levels prior to the study, HbA1c, and glycated hemoglobin estimated (eA1c) obtained from blood glucose levels using the equation from the ADAG study^16^.

Patients were divided into three groups based on G6PD values: *deficient, intermediate, and nondeficient*. In each group, a comparison of the average eA1c and HbA1c values was performed. Then, the difference between the eA1c and HbA1c values of each patient and the mean of the differences (MD) of all patients was calculated within the three groups (deficient, intermediate, nondeficient). Finally, a comparison of the MD values between groups was performed.

HbA1c was measured on capillary blood samples using the DCA Vantage instrument, Siemens. For G6PD measurement, the ratio between *glucose 6 phosphate dehydrogenase/6 phosphogluconic dehydrogenase* was utilized. Subjects with values < 0.10 were considered deficient, with values 0.10–0.85 intermediate, and with values > 0.85 nondeficient.

The Shapiro test was used to assess the normal distribution of variables. Continuous distributed variables are expressed as the mean and standard deviation. The paired t test was used to compare eA1c and HbA1c within G6PD groups. One-way analysis of variance with Tukey’s post hoc test was used to compare eA1c, HbA1c, and MD between G6PD groups. Values of *p* < 0.05 were considered significant. Statistical analysis was performed with the SPSS 28.0 package.

### Ethical approval

The study was performed in accordance with the ethical standards as laid down in the 1964 Declaration of Helsinki and its later amendments or comparable ethical standards and was approved by the AOU Cagliari Ethics Committee.

### Informed consent

Informed consent was obtained from all study participants.

## Results

Seventy-four subjects (50 males) with T1D and a mean age of 12.9 ± 3.5 years (range 3–21 years) were studied. Based on the G6PD value, 33 subjects were deficient (30 hemizygous males and 3 homozygous females), 8 were intermediate (all heterozygous females), and 33 subjects were nondeficient (20 males and 13 females).

In the 90 days prior to the study, the number of daily capillary blood glucose measurements was 5.7 ± 1.2, without differences among the three groups. Table 1. All patients performed at least four daily measurements. Twenty-one (64%) had five measurements, and 6 (18%) had six measurements. The meters used were Accu-Chek Guide Roche and OneDay Ascensia.

**Table 1:** Demographic and metabolic characteristics of patients.

	All	Deficient	Intermediate	Nondeficient	*p* value
Number	74	33	8	33	
Boys/girls n	50/24	30/3	0/8	20/13	
Age years	12.9 ± 3.5	13.8 ± 3.4	11.6 ± 4.6	12.4 ± 3.2	0.117
Glucose measurements	5.7 ± 1.2	5.4 ± 1.1	5.5 ± 1.3	6.0 ± 1.2	0.071
Glycemia mg/dL	170 ± 34	178 ± 32	155 ± 30	165 ± 35	0.140

Data are expressed as mean and standard deviation.

Glucose measurements: average number of daily capillary blood glucose measurements in the 90-day prior to the study.

Glycemia: average blood glucose levels in the 90-day prior to the study.

One-way analysis of variance was used to compare G6PD groups.

The eA1c, HbA1c, and MD values in the three groups are shown in Table 2. In deficient group, the eA1c values were significantly higher than the HbA1c values (7.8 ± 1.1 vs. 6.3 ± 0.8, *p* < 0.001). All deficient subjects had eA1c values higher than HbA1c values except in one case in which they were equal. In the other two groups (intermediate and nondeficient) there were no differences between eA1c and HbA1c.

**Table 2:** eA1c, HbA1c, and MD values in deficient, intermediate and nondeficient subjects.

	eA1c	HbA1c	MD	*p* value	Attributable difference
Deficient %	7.8 ± 1.1	6.3 ± 0.8*	1.5 ± 0.9**	**< 0**.**001**	1.3%
mmol/mol	62 ± 12	45 ± 9	16 ± 10		
Intermediate %	7.1 ± 1.1	6.5 ± 0.7	0.5 ± 0.7	0.075	0.3%
mmol/mol	54 ± 12	48 ± 8	5 ± 8		
Nondeficient %	7.4 ± 1.2	7.2 ± 0.9	0.2 ± 0.7	0.066	
mmol/mol	57 ± 13	55 ± 10	2 ± 8		

Data are expressed as mean and standard deviation.

eA1c: glycated hemoglobin estimated.

HbA1c: glycated hemoglobin.

MD: Mean of differences between eA1C and HbA1c.

The paired t test was used to compare eA1c and HbA1c within G6PD groups.

Significant values are in bold.

One-way analysis of variance with Tukey’s post hoc test was used to compare eA1C, HbA1c, and MD between G6PD groups.

*Tuckey test Deficient versus Nondeficient *p* = 0.009.

** Tuckey test Deficient versus Nondeficient *p* < 0.001 Deficient versus Intermediate *p* < 0.001.

The MD values between the three groups were significantly different. In deficient patients, MD values were higher than those in intermediate (1.5 ± 0.9 vs. 0.5 ± 0.7 *p* < 0.001) and in nondeficient (1.5 ± 0.9 vs. 0.2 ± 0.7 *p* < 0.001). No difference was found between intermediate and nondeficient subjects.

The eA1c, HbA1c, and MD values did not change, including in the calculation only G6PD-deficient subjects who had at least 5 (64%) or six (18%) capillary blood glucose levels measured per day (data not shown). In G6PD-deficient subjects, there was a positive correlation between eA1C and MD (r = 0.74, *p* < 0.001). However, when dividing them according to HbA1c values (< 6, 6–7, > 7%), there were no significant differences in MD. Table 3. In G6PD-deficient subjects, no correlation was found between age and MD.

**Table 3:** Characteristics of G6PD-deficient patients divided by HbA1c values.

	HbA1c %			*p* value
	< 6.0	6.0–7.0	> 7.0	
Number	12	16	5	
Boys/girls number	12/0	15/1	3/2	
Age years	14.4 ± 2.7	13.8 ± 4.3	12.7 ± 1.1	0.665
Glucose measurements	5.2 ± 0.9	5.5 ± 1.2	5.4 ± 1.2	0.851
Glycemia mg/dL	164 ± 28	176 ± 26	219 ± 34	**0**.003*
eA1c %	7.4 ± 1.0	7.7 ± 0.9	9.3 ± 1.2	**0**.003**
mmol/mol	57 ± 11	61 ± 10	78 ± 13	
HbA1c %	5.6 ± 0.2	6.4 ± 0.3	7.7 ± 0.4	< **0**.001***
mmol/mol	38 ± 2	48 ± 3	61 ± 4	
MD %	1.7 ± 1.0	1.4 ± 0.8	1.5 ± 1.3	0.621
mmol/mol	19 ± 11	15 ± 9	16 ± 14	

Data are expressed as mean and standard deviation.

Glucose measurements: average number of daily capillary blood glucose measurements in the 90-day prior to the study.

Glycemia: average blood glucose levels in the 90-day prior to the study.

eA1c: glycated hemoglobin estimated.

HbA1c: glycated hemoglobin.

MD: Mean of differences between eA1C and HbA1c.

One-way analysis of variance with Tukey’s post hoc test was used to compare HbA1c groups.

*Tuckey test HbA1c < 6 versus HbA1c > 7 *p* < 0.001 HbA1c 6–7 versus HbA1c > 7 *p* = 0.008.

**Tuckey test HbA1c < 6 versus HbA1c > 7 *p* = 0.008 HbA1c 6–7 versus HbA1c > 7 *p* = 0.007.

*** Tuckey test HbA1c < 6 versus HbA1c 6–7 *p* < 0.001 HbA1c < 6 versus HbA1c > 7 *p* < 0.001 HbA1c 6–7 versus HbA1c > 7.

*p* < 0.001.

Significant values are in bold.

## Discussion

Our study confirms that G6PD deficiency affects HbA1c values in children and adolescents with T1D, both in deficient subjects (hemizygous males and homozygous females) and, to a much lesser extent, in intermediate subjects (heterozygous females). It also allows us to quantify the correction to be made on the HbA1c values. In deficient subjects, there is an average reduction in HbA1c attributable to enzyme deficiency of 1.3% (14 mmol/mol) and in intermediate subjects of 0.3% (3 mmol/mol).

These findings may have clinical relevance in several situations. In Sardinia, the prevalence of G6PD deficiency in the general population is 11%^17^. The disorder is common in Africa, Asia, the Middle East, Latin America, and the Mediterranean^6^. Global migration means that, even in countries where G6PD deficiency is very rare, diabetes specialists are more likely to see patients with T1D and G6PD deficiency.

Prevention of microvascular complications (retinopathy, nephropathy, neuropathy) requires optimal metabolic control from the onset of the disease; therefore, HbA1c monitoring is still important in the management of patients with T1D. The glucose monitoring indicator (GMI), a surrogate of HbA1c included in the metrics of continuous glucose monitoring (CGM), does not overlap with HbA1c. Zucchini et al. found that more than one-third of children and adolescents with type 1 diabetes have a clinically significant discordance between GMI and HbA1c^18^. In children and adults without diabetes, Shah et al. found that the mean GMI was 0.59% higher than the laboratory HbA1c^19^. In patients with diabetes and G6PD deficiency, GMIs are expected to be higher than HbA1c values because they reflect subcutaneous glucose values following plasma glucose. This may be a contributing factor to the difference between GMI and HbA1c.

Furthermore, there is another condition in which G6PD deficiency can be responsible for a delay in the diagnosis of diabetes. It concerns children with stress-induced hyperglycemia, for example infection diseases, and those with occasional mild hyperglycemia. T1D has a long preclinical course in which the patient may be asymptomatic with mild hyperglycemia (100–125 mg/dL) and a mild increase in HbA1c values (5.8–6.4%)^20^. In people with G6PD deficiency, the finding of mild hyperglycemia may be associated with falsely normal HbA1c values, delaying the diagnosis, and leading to a later risk of ketoacidosis. Even in patients with monogenic diabetes (MODY), the initial presentation is often with mild and inconstant fasting hyperglycemia and HbA1c values in the range of prediabetes (5.8–6.4%). Again, G6PD deficiency may be responsible for a delay in diagnosis.

A limitation of our study is that, because of its retrospective nature, it was not possible to determine whether patients with G6PD deficiency had infectious diseases or were taking medications that can cause hemolysis in the weeks prior to HbA1c measurements. During hemolytic crisis, reticulocytosis can lead to a G6PD dosage close to normal. This possibility in our sample of children with T1D and G6PD deficiency is very unlikely because hemolysis due to infectious diseases is rare and manifests with very obvious clinical signs. In addition, patients with T1D and G6PD deficiency are always carefully instructed on which medications to exclude.

In conclusion, our study confirms that HbA1c is not a reliable tool to assess metabolic control in patients with G6PD deficiency and T1D or prediabetes. In countries with a high prevalence of G6PD deficiency, G6PD measurement should be included in routine examinations both at the onset of diabetes and for the investigation of mild hyperglycemia.

## Data Availability

The datasets analyzed during the current study are available from the corresponding author on reasonable request.
